# Joule Heating Effects on Transport-Induced-Charge Phenomena in an Ultrathin Nanopore

**DOI:** 10.3390/mi11121041

**Published:** 2020-11-26

**Authors:** Zhixuan Wang, Wei-Lun Hsu, Shuntaro Tsuchiya, Soumyadeep Paul, Amer Alizadeh, Hirofumi Daiguji

**Affiliations:** Department of Mechanical Engineering, The University of Tokyo, Bunkyo-ku, Tokyo 113-8656, Japan; wangzx@thml.t.u-tokyo.ac.jp (Z.W.); tsuchiya.s@hnl.t.u-tokyo.ac.jp (S.T.); soumyadeep.paul@thml.t.u-tokyo.ac.jp (S.P.); aalizadeh@thml.t.u-tokyo.ac.jp (A.A.)

**Keywords:** transport-induced-charge, nanofluidics, electrokinetics, Joule heating

## Abstract

Transport-induced-charge (TIC) phenomena, in which the concentration imbalance between cations and anions occurs when more than two chemical potential gradients coexist within an ultrathin dimension, entail numerous nanofluidic systems. Evidence has indicated that the presence of TIC produces a nonlinear response of electroosmotic flow to the applied voltage, resulting in complex fluid behavior. In this study, we theoretically investigate thermal effects due to Joule heating on TIC phenomena in an ultrathin nanopore by computational fluid dynamics simulation. Our modeling results show that the rise of local temperature inside the nanopore significantly enhances TIC effects and thus has a significant influence on electroosmotic behavior. A local maximum of the solution conductivity occurs near the entrance of the nanopore at the high salt concentration end, resulting in a reversal of TIC across the nanopore. The Joule heating effects increase the reversal of TIC with the synergy of the negatively charged nanopore, and they also enhance the electroosmotic flow regardless of whether the nanopore is charged. These theoretical observations will improve our knowledge of nonclassical electrokinetic phenomena for flow control in nanopore systems.

## 1. Introduction

Ultrathin nanopores are artificial apertures of nanoscale dimensions on sub-100 nm inorganic membranes [1]. In comparison with biological pores on protein membranes, these pores possess unrivaled advantages of flexibility in dimensions, mechanical robustness, and chemical stability, and hence stimulate the development of frontier technologies for versatile applications [2]. Utilizing a two-dimensional (2D) material, monolayer molybdenum disulfide, Feng et al. [3] demonstrated extraordinary energy conversion performance of reverse electrodialysis when converting a salinity concentration difference into electricity. Paul et al. [4] blueprinted a nanopore-based bubble emitter for cooling applications in closely packed electronic components in response to the rising demands of high-performance computers. As viruses electro-migrate through nanopores, their structures and charge conditions can be identified by detecting the ionic current variations [5], which is imperative for medical diagnosis in society after COVID-19.

To further pursue the improved performance and accuracy of these applications, it is vital to examine the ion and flow behaviors in ultrathin nanopores from a fundamental perspective. When these nanopores are immersed in an electrolyte solution, the positively/negatively charged surface results in the selectivity of ions that renders higher penetration of anions/cations over their counterparts, as long as an electric potential difference is present across the membrane. This ionic selectivity causes a slight salinity concentration imbalance between the two sides of the membrane, known as ion concentration polarization (ICP) effects [6]. In other words, the axial electric potential gradient induces an axial concentration gradient due to the radial electric field induced by the surface charge. The magnitude of these gradients is determined by the concentration of bulk salinity, applied electrical potential difference, and thickness of the membrane. As the membrane becomes considerably thin, the interaction between the strong electric field and the steep concentration gradient promotes local ion separation, resulting in a net space charge outside the electrical double layer (EDL). This phenomenon, known as transport-induced-charge (TIC) effects [7], disturbs the ionic distributions in the nanopores and considerably influences the electroosmotic behavior.

TIC effects due to the coexistence of an electric field and a concentration gradient were theoretically reported by Rademaker et al. [8] in an electrochemical system of lithium-ion batteries. However, although the induced charge could occur in their system, it was negligible compared to the bulk ion concentrations. On the contrary, in ultrathin nanopores, both the electric field and the concentration gradient are focused and amplified within a nanoscale dimension, yielding significant TIC effects that influence nanopore electrokinetics. Hsu et al. [7] investigated the behavior of electroosmotic flow (EOF) caused by TIC (TIC EOF) via computational simulation. The results indicated that the TIC EOF is dominant at the high applied electric potential difference (~1 V) compared with the conventional electroosmotic flow originating from the EDL (EDL EOF). Thus, the TIC EOF can reverse the flow direction when the applied electric potential difference exceeds a certain critical value. This unique phenomenon properly explained the anomalous DNA translocation behavior observed by molecular dynamics in a previous study [9], in which a threshold voltage was required to drive DNA molecules through a 2D nanopore in order to overcome the opposing EDL EOF.

In the model of Hsu et al. [7], it was assumed that the system maintained an isothermal condition and the simultaneous Joule heating effects produced by the applied electric field were not considered. However, this assumption cannot remain valid for the cases at high applied voltages. For instance, Nagashima et al. [10] demonstrated homogeneous bubble formation activated by superheating in nanopores attributed to Joule heating effects when an extra-high voltage was imposed (e.g., 8 V). The existence of a non-uniform temperature results in overlapping of triple effects (temperature gradient, salt concentration gradient, and electrical field) within a small distance in ultrathin nanopores, which could lead to intricate electrokinetic behavior. In this study, we theoretically investigate the coupling of Joule heating effects with TIC phenomena via computational simulation. Thus, the variation in the TIC in response to the localized temperature rise can be controlled. In addition, the impact of TIC on the electroosmosis behavior is evaluated, which may enrich our understanding of complex electrokinetic phenomena in ultrathin nanopores.

## 2. Computational Modeling

**Figure 1:**
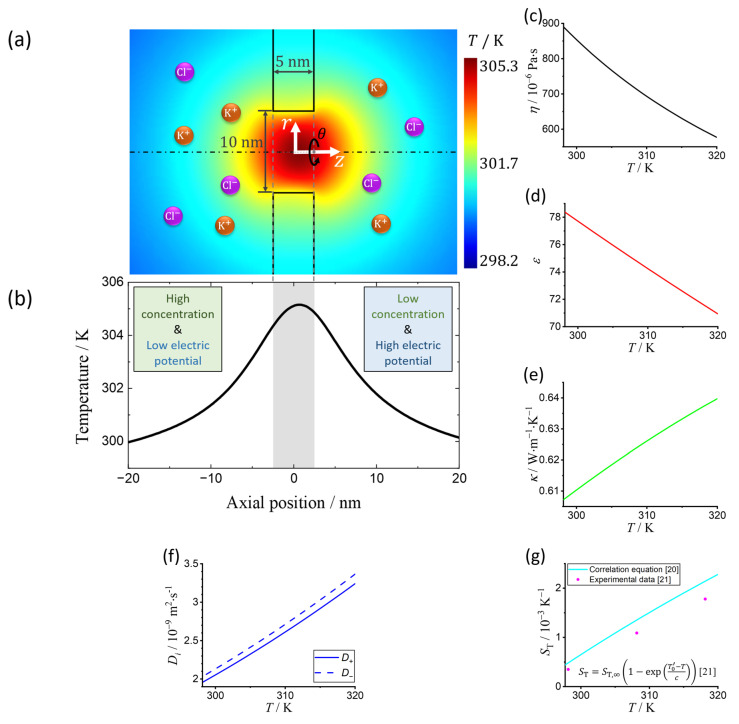
(**a**) Schematic illustration of a 10 nm (in diameter) nanopore separating two KCl solutions with different concentrations. The concentration of the solution on the left side of the reservoir is higher than that on the right side, whereas the electric potential on the right side is higher than that on the left side. The temperature inside the nanopore has a significant increase due to Joule heating effects. (**b**) Typical axial temperature distribution in an ultrathin nanopore with Joule heating effects. (The shaded part indicates the position of the nanopore.) Relations between the (**c**) viscosity η of pure water, (**d**) static dielectric constant ε, and (**e**) thermal conductivity κ and temperature T at 0.1 MPa. Temperature responses of the (**f**) diffusivity $D_{i}$, and (**g**) Soret coefficient $S_{T}$ of aqueous KCl, where the subscript i of “+,−” denotes the cation $K^{+}$ and anion ${\mathrm{Cl}}^{-}$, respectively.

A steady-state model of Joule heating effects on TIC phenomena in an ultrathin nanopore in a 2D cylindrical coordinate is investigated. As illustrated in Figure 1a, a nanofluidic system with a silicon nitride (SiN) nanopore (L=5 nm thick and $D_{p}=10\;\mathrm{nm}$ in diameter) filled with asymmetrically concentrated potassium chloride (KCl) aqueous solutions from each side is considered [11,12,13]. Then, an external electric field is applied parallelly to the salt concentration gradient. The midpoint of the nanopore is set as the origin *O*, an arbitrary radial direction as the polar axis r, and the central axis of the nanopore as the cylindrical axis z. Accordingly, a 2D cylindrical coordinate system $\left(r,z\right)$ is employed, and the system is symmetric with respect to the azimuthal θ-direction.

By considering mass conservation, we adopt the continuity equation for the solution at steady state:

(1) ∇⋅v=0,

where v is the velocity vector of the solution. Note that although the solution density ρ depends on the solute concentration and temperature, the concentration of water molecules (~55.6 M) is considerably higher than the solute concentration, making the density barely change with the amount of KCl dissolved. In addition, between 25–99 °C the density variation of pure water is less than 4% [14]. Thus we assume that ρ is constant in the system (i.e., incompressible fluid), equal to the density of pure water.

The typical axial temperature distribution in this flow system is shown in Figure 1b. Due to the huge difference between the concentrations of solvent and solute, the temperature-dependent solution properties are also replaced by pure water properties as shown in Figure 1c–e, the solution properties, including the viscosity η, static dielectric constant ε, and thermal conductivity κ, are considered from previous experimental results of pure water, which are valid between 273.15–383.15 K [15] (detailed information is provided in the Supplementary Materials Section S3). The specific isobaric heat capacity $c_{p}$ is regarded as constant because its variation with temperature is less than 0.06% between 298.15–320 K [15], covering the temperature range in this study.

A modified steady-state Navier–Stokes equation considering the electric force on the electrolyte solution is used:

(2) $$\rho v\cdot \nabla v=-\nabla p+\nabla \cdot \left(\eta \left(\nabla v+{\left(\nabla v\right)}^{T}\right)\right)-\rho_{e}\nabla \phi -\frac{1}{2}{\left|-\nabla \phi \right|}^{2}\nabla \left(\varepsilon \varepsilon_{0}\right),$$

where p, $\rho_{e}$, ϕ, $\varepsilon_{0}$ are the pressure, space charge density, electric potential, and absolute dielectric permittivity of classical vacuum, respectively. The variation in viscosity η [16] due to the local electric field is not considered. The terms $-\rho_{e}\nabla \phi$ and $-\frac{1}{2}{\left|-\nabla \phi \right|}^{2}\nabla \left(\varepsilon \varepsilon_{0}\right)$ are the electric body force on the free charges distributed in the solution and the dielectric force on the solvent due to the variation of ε, respectively [17].

The steady-state ion distributions can be described by the Nernst–Planck equation [18], where the subscript i of “+, −” denotes the cation $K^{+}$ and anion ${\mathrm{Cl}}^{-}$, respectively:

(3) $$\nabla \cdot \left(vn_{i}-D_{i}\nabla n_{i}-\mu_{i}n_{i}\nabla \phi -D_{T,i}n_{i}\nabla T\right)=\nabla \cdot \left(J_{i,\mathrm{adv}}+J_{i,\mathrm{diff}}+J_{i,\mathrm{cond}}+J_{i,\mathrm{therm}}\right)=0,$$

where, $n_{i}$, $D_{i}$, $\mu_{i}$, and $D_{T,i}$ are the molar concentration, diffusivity, mobility, and thermal diffusion coefficient of ions, respectively. $J_{i,\mathrm{adv}}$, $J_{i,\mathrm{diff}}$, $J_{i,\mathrm{cond}}$, and $J_{i,\mathrm{therm}}$ represent the advection, diffusion, conduction, and thermodiffusion ionic fluxes, respectively. According to the Stokes–Einstein equation, $D_{i}=D_{i,0}\frac{\eta_{0}}{T_{0}}\frac{T}{\eta }$, where $D_{i,0}$ and $\eta_{0}$ are the ionic diffusivity and the viscosity of water at temperature $T_{0}=298.15\;K$, respectively. We use $\;D_{+,0}=1.957\times 10^{-9}\;m^{2}\cdot s^{-1}$ for $K^{+}$ and $D_{-,0}=2.032\times 10^{-9}\;m^{2}\cdot s^{-1}$ for ${\mathrm{Cl}}^{-}$ [14]. $J_{i,\mathrm{cond}}$ generated from electrochemical migration is proportional to the ionic mobility $\mu_{i}=\pm \frac{eD_{i}}{k_{B}T}$, where e and $k_{B}$ are the elementary charge and Boltzmann’s constant, respectively. The variation of $D_{i}$ as a function of temperature is shown in Figure 1f. $J_{i,\mathrm{therm}}$ arises from the Soret effect due to the presence of the temperature gradient, also known as thermodiffusion [19]. The relation between the thermal diffusion coefficient and diffusivity is described as $S_{T}=\frac{D_{T,i}}{D_{i}}$, where $S_{T}$ is the Soret coefficient, which is usually determined empirically. When $S_{T}>0$, thermodiffusion is from hot to cold regions, whereas $S_{T}<0$ denotes thermal migration of ions from cold to hot regions. Here, we adopt an empirical correlation valid between 280 K and 340 K from a previous study, in which $S_{T}=S_{T,\infty }\left(1-\exp\left(\frac{T_{0}^{’}-T}{c}\right)\right)$, where $S_{T,\infty }=0.005\;K^{-1}$, $T_{0}^{’}=283\;K$, and c=102 K [20], measured at the KCl concentration of 0.5 M. Note that these parameters are largely insensitive to the solute concentration at the currently investigated concentration level [20,21]; hence, we consider these parameters to be constant. In this regard, the relation between the Soret coefficient and temperature is plotted in Figure 1g.

The electric potential distribution is described by the Poisson equation as below:

(4) $$\nabla \cdot \left(\varepsilon \nabla \phi \right)=-\frac{\rho_{e}}{\varepsilon_{0}}=-\frac{eN_{A}\left(n_{+}-n_{-}\right)}{\varepsilon_{0}},$$

where $N_{A}$ is the Avogadro constant.

The steady-state energy balance for the liquid electrolyte solution is described by Equation (5):

(5) $$\rho c_{p}v\cdot \nabla T=\nabla \cdot \left(\kappa \nabla T\right)-j_{I}\cdot \nabla \phi ,$$

where $j_{I}$ is the current density. The term $-j_{I}\cdot \nabla \phi$ represents the heat source caused by the Joule heating effects and $j_{I}$ can be obtained by the ionic flux as $j_{I}=e\left(J_{+,\mathrm{diff}}+J_{+,\mathrm{cond}}+J_{+,\mathrm{therm}}\right)-e\left(J_{-,\mathrm{diff}}+J_{-,\mathrm{cond}}+J_{-,\mathrm{therm}}\right)$, in which the advection ionic flux terms are not considered because they do not contribute to the relative velocity between the ions and the liquid [22], despite their role on the current. It can be seen that the currents $I_{+}$ formed by positive ions and $I_{-}$ by negative ions of the system are also composed of four parts, $I_{\pm }=I_{\pm ,\mathrm{adv}}+I_{\pm ,\mathrm{diff}}+I_{\pm ,\mathrm{cond}}+I_{\pm ,\mathrm{therm}}$, in which the four terms on the right represent the contribution of the corresponding integrated ionic flux on the cross-sectional area of the system; for example $I_{\pm ,\mathrm{cond}}=\pm eN_{A}\iint J_{\pm ,\mathrm{cond}}\cdot ndS$, in which the direction of the unit surface normal n is the same as the positive direction of the system. Viscous dissipation is not considered in this system because of its limited influence on the temperature rise compared to Joule heating effects (detailed information is provided in the Supplementary Materials Section S2).

For the silicon nitride thin layer, the electric potential distribution is not considered, and the steady-state energy conservation equation is expressed as:

(6) $$\nabla^{2}T=0.$$

**Figure 2:**
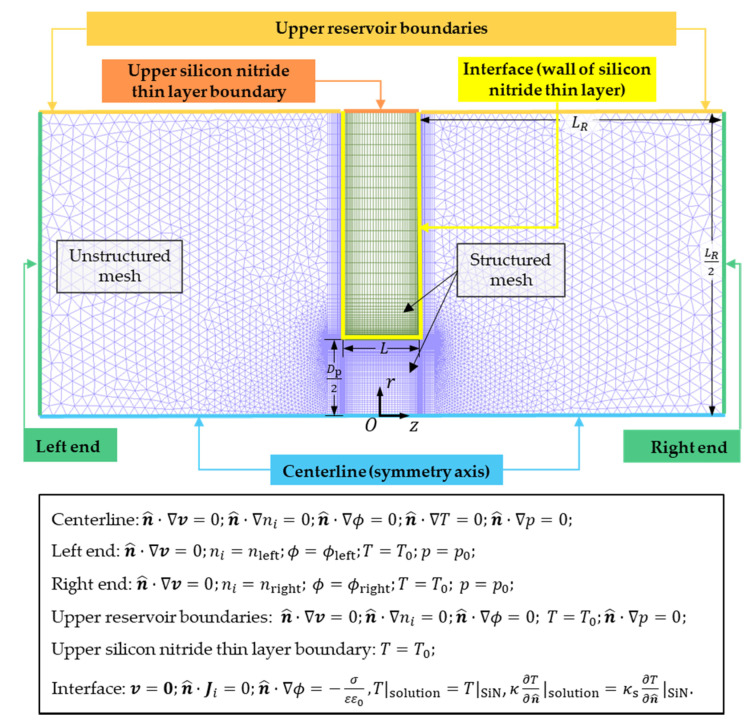
A hybrid computational mesh consists of unstructured and structured grids. Boundary conditions are listed in the underneath table. Note that the actual computational domain used is much larger to achieve mesh independence (see the Supplementary Materials Section S1).

Figure 2 shows the axisymmetric 2D model with boundary conditions. $\hat{n}$ is the normal unit vector pointing outside ($\hat{n}$ is pointing toward the layer on the interface of the solution and the silicon nitride thin layer). On the centerline (along the z-axis), the pressure, velocity, concentration, electric potential, and temperature are satisfied with symmetry boundary conditions. At the far ends of the two reservoirs, the flow is considered to be fully developed with a constant concentration, temperature, electric potential, and pressure. A concentration bias ∆n=0.4 M is applied to the two reservoirs such that the solution concentrations at the left and right far ends are $n_{\mathrm{left}}=n_{0}+\frac{∆n}{2}=1.2\;M$ and $n_{\mathrm{right}}=n_{0}-\frac{∆n}{2}=0.8\;M$, respectively. Here, $n_{0}=1\;M$ is the average molar concentration of the solution. The temperature at the left and right end is maintained at $T_{0}=298.15\;K$. An external electric potential difference ∆ϕ is applied between the right and left end of the system and $∆\phi =\phi_{\mathrm{right}}-\phi_{\mathrm{left}}$, where $\phi_{\mathrm{right}}=\frac{∆\phi }{2}$ and $\phi_{\mathrm{left}}=-\frac{∆\phi }{2}$ are the electric potential of the right and left ends, respectively. Experimentally, one end of the solution is normally connected to ground as a reference electric potential. However, given that the electric potential is a relative quantity, the selection of the reference potential would not alter the physical phenomena. One can interpret that the reference electric potential taken here as the ground potential plus $\frac{∆\phi }{2}$ (in the case when the low potential end is grounded). On the upper boundaries, the temperature is fixed at $T_{0}$ and symmetry boundary conditions are applied for other unknowns for both reservoirs. At the interface between the silicon nitride thin layer and the solution, the nonslip boundary condition is adopted, the ion flux is zero along $\hat{n}$, and the temperature and heat flux are continuous. The thermal conductivity of silicon nitride $\kappa_{s}=3.2\;W\cdot m^{-1}\cdot K^{-1}$ is obtained from [23]. The surface charge density σ on the silicon nitride thin layer is related to the local electric field as [24]:

(7) $$\hat{n}\cdot \nabla \phi =-\frac{\sigma }{\varepsilon \varepsilon_{0}}.$$

Due to the deprotonation reaction of silanol groups on the surface, σ can be affected by temperature due to the variation of the reaction constant. In the literature, the surface charge density at the silica/water interface subject to silanol groups at different temperature has been investigated both theoretically and experimentally. Close to room temperature, a previous theoretical study based on an electrical quad-layer model coupled with temperature effects [25] shows that surface charge density increases less than approximately 6% when the temperature is elevated by 10 K. Similarly, using sum frequency generation measurements, recent experimental results demonstrate that the increase of surface charge density due to temperature effects near room temperature is marginal [26]. Therefore, according to the temperature range considered, we neglect the variation of surface charge density due to thermal effects in our modeling. However, a complex surface model may be needed for higher temperatures (>75 °C).

Continuum dynamics can describe the transport phenomena of ions in nanofluidic channels when the length scale is above 5 nm [27]. The previous results of molecular dynamics show that the temperature gradient is continuous in the liquid phase at the quasi-atomic scale [28,29], thus verifying the continuous assumption including the temperature field inside our system. On this account, we solve the governing equations by numerical simulation, which is discretized in an implicit finite volume formulation [30] on a hybrid mesh consisting of structured and unstructured grids. The length $L_{R}$ of the reservoir in the computational domain is 600 nm, which can provide sufficiently accurate results that are not sensitive to the size of the reservoir and remain computationally efficient. Even if $L_{R}$ is increased to 1000 nm with a larger computational domain and more and denser mesh grids, the relative difference of the simulation results between these two cases is smaller than 2.5% (detailed information is provided in the Supplementary Materials Section S1). The total cell number of the mesh is 149,004, including 100,086 unstructured triangles and 48,918 structured quadrangles.

## 3. Results and Discussion

### 3.1. TIC Phenomena in the Presence of Joule Heating

**Figure 3:**
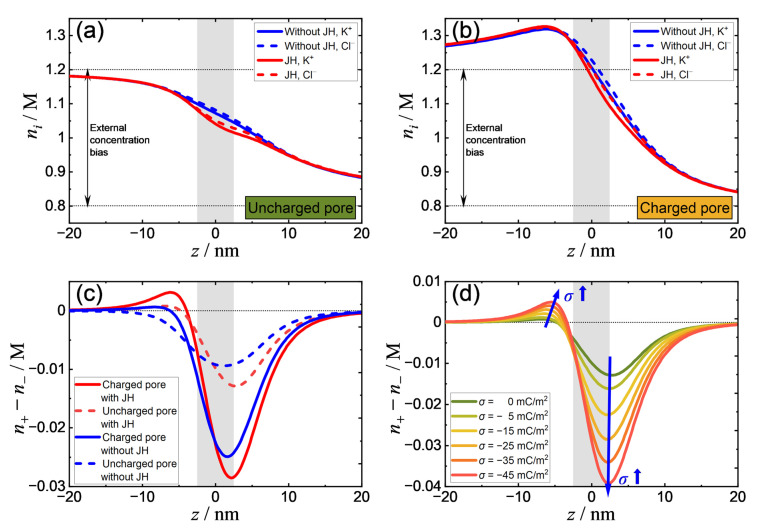
(**a**) Ion concentration distribution of cation and anion in an uncharged nanopore with/without Joule heating; (**b**) ion concentration distribution of cation and anion in a negatively charged nanopore with/without Joule heating, where $\sigma =-25\;\mathrm{mC}\cdot m^{-2};$ (**c**) induced charge concentration in four different cases: Uncharged/negatively charged nanopore with/without Joule heating effects; (**d**) effect of surface charge density on transport-induced-charge (TIC). (The “JH” in the legend denotes Joule heating.)

We compared TIC phenomena in four different cases: (i) An uncharged pore without Joule heating, (ii) an uncharged pore with Joule heating, (iii) a negatively charged pore without Joule heating, and (iv) a negatively charged pore with Joule heating. In all cases, a solution concentration difference of ∆n=0.4 M and an electric potential difference of ∆ϕ=2.0 V are applied across a 10 nm (diameter) nanopore. When Joule heating is considered, the ionic concentrations become slightly lower in the nanopore as shown in Figure 3a,b. As the surface charge exists, the imbalance of the salt concentration becomes more significant due to ICP effects [6]. Figure 3c shows the ionic concentration difference $n_{+}-n_{-}$ (proportional to the TIC concentration) along the centerline. Except for Case (i), where the TIC is nearly symmetric to the center of the nanopore, positive TIC is induced at the entrance of the nanopore, opposite to the negative TIC inside the nanopore, although the positive TIC concentration is rather low for Case (iii), at $6.4\times 10^{-4}\;M$. Note that when Joule heating or surface charge is present, this asymmetry becomes more prominent. The positive TIC reaches a maximum when both the surface charge and Joule heating effects are considered. As shown in Figure 3d, when the surface charge is increased, the amount of both the positive and negative TIC increases.

### 3.2. Mechanism of TIC Phenomena

**Figure 4:**
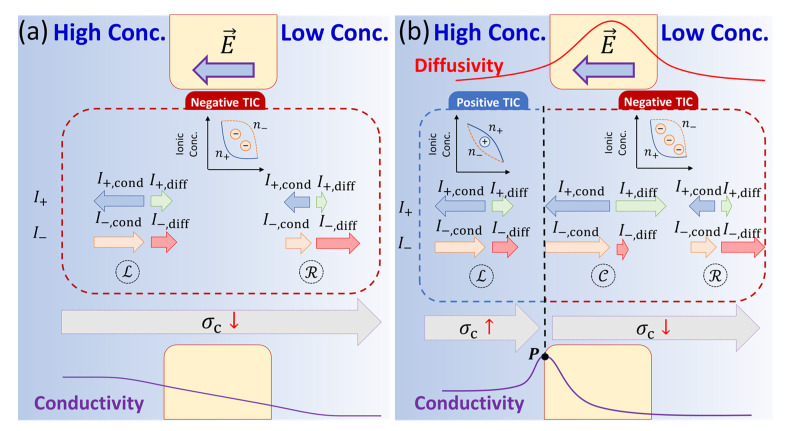
Schematic (not to scale) explanation of (**a**) negative TIC in an isothermal nanopore under the influence of only ion concentration gradient, and (**b**) combination of positive and negative TIC in a nanopore under the dual influence of ion concentration gradient and temperature hotspot formed as a result of Joule heating.

In this section, we explore the underlying mechanism for the induction of positive TIC and the amplification of the negative TIC identified in Section 3.1. We analyze dual influence of the ion concentration gradient and temperature hotspot on the TIC distribution. TIC is primarily subject to the spatial variation of ionic conductivity, which in turn depends on the ion concentration and ionic diffusivity. The scenario where the TIC is merely governed by the salt concentration variation in the absence of thermal gradients [7] is summarized in Figure 4a. We investigate the TIC phenomenon by comparing the conduction currents, $I_{\mathrm{cond}}$, developed in response to the electric field and the diffusion currents, $I_{\mathrm{diff}}$, developed in response to the ion concentration gradient. Owing to the higher ion concentration near the nanopore inlet in Region L, higher magnitudes of $I_{+,\mathrm{cond}}$ (blue arrows) and $I_{-,\mathrm{cond}}$ (orange arrows) are developed compared to the outlet in Region R. To balance this asymmetry, non-uniform diffusion currents are established to ensure the conservation of ions, such that $I_{+,\mathrm{diff}}$ decreases from the inlet to the outlet, while $I_{-,\mathrm{diff}}$ increases from the inlet to outlet (Figure 4a). The variation in the diffusion current directly affects the ion concentrations according to Fick’s law ($\nabla n_{i}\propto I_{i,\mathrm{diff}}$). A higher cation diffusion current at the inlet indicates a larger concentration gradient at the inlet compared to the outlet. In contrast, a lower anion diffusion current at the inlet indicates a smaller concentration gradient at the inlet compared to the outlet. This leads to a convex/concave distribution of the cations/anions, effectively inducing a negative charge inside the nanopore. In summary, we find that negative TIC appears when the electric field and conductivity gradient are parallel.

As the influence of the concentration bias is coupled with Joule heating, the monotonic decrease in ionic conductivity across the nanopore does not maintain. Because of the thermal hotspot at the nanopore center (Figure 1a), an ionic diffusivity peak appears near the nanopore center (see the red curve in Figure 4b). In conjunction with the externally applied concentration bias, this results in a conductivity peak P near the nanopore inlet (the violet curve in Figure 4b). From the peak P towards the outlet, the conductivity decreases monotonically in Regions C and R, and thus, the development of $I_{\mathrm{cond}}$ and $I_{\mathrm{diff}}$ for both cations and anions follow the same tendency as shown in Figure 4a. This gives rise to the presence of negative TIC of higher magnitude, as the conductivity gradient is high due to the comparatively high diffusivity inside the nanopore compared to the outlet.

In the region to the left of P, attributed to the conductivity increase from the bulk solution towards the nanopore inlet, $I_{\mathrm{cond}}$ increases accordingly. This yields a lower $I_{+,\mathrm{diff}}$ in Region L compared to Region C and a higher $I_{-,\mathrm{diff}}$ in Region L compared to Region C (Figure 4b), ensuring the ion conservation. Similar to that in the negative TIC region, this asymmetric diffusion current indicates a concave/convex distribution of the cations/anions, effectively inducing a positive charge in Region L. Thus, for Figure 4b, we find that negative/positive TIC appears when the electric field and conductivity gradient are in the same/opposite direction.

The identified TIC phenomena can be further supported by the Poisson and Nernst–Planck equations. According to the conservation of ions, Equation (8) is satisfied at steady state. Note that the contribution of ionic conduction flux is much larger than others (e.g., for Case (iv) in Section 3.1, the maximum magnitude of the conduction flux for $K^{+}$ is approximately 6 times higher than that of the advection flux, 141 times of the diffusion flux, and $8.9\times 10^{3}$ times of the thermodiffusion flux, and for ${\mathrm{Cl}}^{-}$ it is 7, 166, and $9.1\times 10^{3}$ times higher, respectively). Thus, we obtain:

(8) $$0=\frac{\partial \rho_{e}}{\partial t}=-\nabla \cdot j_{I}≅-\nabla \cdot j_{I,\mathrm{cond}},$$

where $j_{I,\mathrm{cond}}=j_{+,\mathrm{cond}}+j_{-,\mathrm{cond}}$ is the net conduction current caused by the ionic conduction flux, which can be expressed as:

(9) $$j_{I,\mathrm{cond}}=\sigma_{c}E,$$

where E is the electric field and $\sigma_{c}$ is the local solution conductivity. Substituting Equation (8) into Equation (9), we derive:

(10) $$\sigma_{c}\nabla \cdot E+\nabla \sigma_{c}\cdot E=0.$$

Considering Poisson’s equation (Equation (4)) and E=−∇ϕ, we get the following expression:

(11) $$\rho_{e}=\varepsilon_{0}\varepsilon \nabla \cdot E+\varepsilon_{0}E\cdot \nabla \varepsilon .$$

Then, combining Equations (10) and (11), the following relation [23] can be derived:

(12) $$\rho_{e}=\varepsilon_{0}\varepsilon E\cdot \left(\frac{\nabla \varepsilon }{\varepsilon }-\frac{\nabla \sigma_{c}}{\sigma_{c}}\right).$$

As demonstrated in Figure 2, the effect of temperature on the dielectric constant ε is relatively insignificant compared with those on the ionic diffusivities. In addition, the effect of salt concentration on ε is negligible. This yields to $\left|\frac{\nabla \varepsilon }{\varepsilon }\right|\ll \left|\frac{\nabla \sigma_{c}}{\sigma_{c}}\right|$ in our systems, as:

(13) $$\rho_{e}\propto -E\cdot \nabla \sigma_{c}.$$

Therefore, as the solution conductivity gradient is in the same direction as the external electric field, negative TIC occurs. In contrast, positive TIC appears when the external electric field is opposite to the local solution conductivity gradient, validating our analysis in Figure 4.

### 3.3. Effects of Joule Heating on Electroosmotic Flow Due to the Presence of TIC

**Figure 5:**
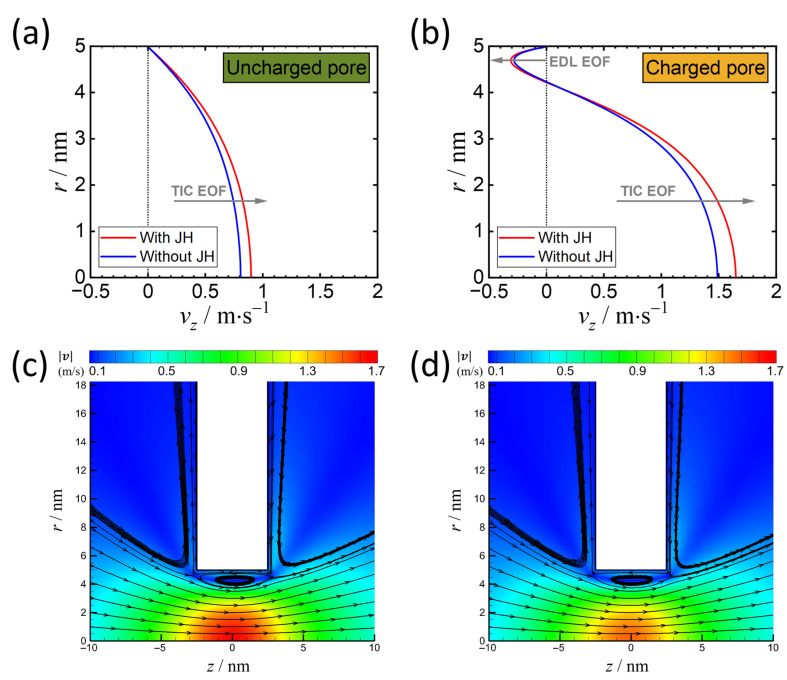
Velocity profiles at z=0 in (**a**) an uncharged and in (**b**) a negatively charged nanopore. Contours of the velocity magnitude and streamlines of the two cases in (**b**) as the surface is charged: (**c**) When considering Joule heating effects, and (**d**) without Joule heating effects. ∆ϕ=2.0 V for all these cases and $\sigma =-25\;\mathrm{mC}\cdot m^{-2}$ for (**b**–**d**).

As seen in Figure 5a, we compare the EOF in the cases with and without Joule heating effects in an uncharged nanopore that demonstrates the enhancement of EOF (by 10–15%) due to the increase in temperature. This EOF acceleration is primarily attributed to the decrease in viscosity (Figure 1c) and the enhancement of TIC concentration (Figure 3c). Although the induction of positive TIC has negative effects on the increase in EOF, the increment of negative TIC is more significant and therefore dominates the EOF behavior. Similarly, as the nanopore is negatively charged (Figure 5b), the EOF due to the surface charge in the vicinity of the nanopore surface also increases, despite the enhancement of TIC effects that drive the solution toward the opposite direction in the middle of the nanopore. The presence of these two types of EOF generates a vortex near the outer boundary of the electric double layer, as shown in Figure 5c,d.

Interestingly, it was found that the surface charge has a synergistic effect with Joule heating in terms of the enhancement of EOF. As seen in Figure 5a,b, the maximum velocity magnitude on the centerline significantly increases when the surface is charged, in spite of the presence of the counterflow on the surface. This indicates that ICP effects are coupled with TIC EOF, which further enlarges the concentration difference across the nanopore, resulting in the enhancement of TIC EOF near the axis caused by the increment of TIC (as shown in Figure 3d).

## 4. Conclusions

Joule heating effects on TIC phenomena in an ultrathin nanopore have been investigated by numerical simulation. The localized temperature enhancement in the nanopore largely alters the fluid properties and ionic concentration distributions. The solution conductivity increases from the high-concentration reservoir to the heated nanopore, resulting in positive TIC due to the opposite directions of the external electric field and the solution conductivity gradient. In contrast, as the conductivity decreases from the nanopore volume to the low-concentration reservoir, the same direction of the external electric field and the solution conductivity gradient leads to negative TIC. The presence of Joule heating enhances the EOF for both uncharged and charged pores. Especially, the increase of EOF due to TIC is more evident in charged nanopores, attributed to the amplified TIC behavior caused by ICP effects. The elucidated mechanism of TIC phenomena provides a useful guide for the control of fluid behavior in ultrathin nanopores.

## Supplementary Materials

The following are available online at https://www.mdpi.com/2072-666X/11/12/1041/s1, S1. Computational domain sensitivity and mesh analysis; S2. comparison of the magnitudes of viscous dissipation and Joule heating; S3. information on correlation equations for pure water at 0.1 MPa.
