# Supplementary material for: Joule Heating Effects on Transport-Induced-Charge Phenomena in an Ultrathin Nanopore
Source: Micromachines (Basel). 2020 Nov 26;11(12):1041. doi: 10.3390/mi11121041 (PMC7761093; doi:10.3390/mi11121041)
Supplement: Supplementary file 1 [file micromachines-11-01041-s001.pdf]

Supplementary Materials for

“Joule Heating Effects on Transport-Induced-Charge Phenomena in an Ultrathin Nanopore”

Zhixuan Wang, Wei-Lun Hsu*, Shuntaro Tsuchiya, Soumyadeep Paul, Amer Alizadeh and Hirofumi Daiguji*

Department of Mechanical Engineering, The University of Tokyo, Bunkyo-ku, Tokyo 113-8656*, Japan*

* Correspondence: wlhsu@thml.t.u-tokyo.ac.jp; Tel.: +81-3-5841-0911 (Wei-Lun Hsu)
daiguji@thml.t.u-tokyo.ac.jp; Tel.: +81-3-5841-6971 (Hirofumi Daiguji)

Table of Contents

1. Computational domain sensitivity and mesh analysis……………………p.2

2. Comparison of the magnitudes of viscous dissipation and Joule heating...p.6

3. Information on correlation equations for pure water at 0.1 MPa…………p.6

1. Computational domain sensitivity and mesh analysis

The size of the computational domain is determined by comparing the simulation results of different computational domain sizes. Because the left and right end boundaries are satisfied with the Dirichlet boundary conditions (fixed boundary condition) for voltage, solution concentration, and temperature (also the up silicon nitride thin layer boundary and up reservoir boundaries for temperature), the size of the computational domain directly affects the simulation results in the nanopore. In addition, different reservoir sizes affect the resistance ratio between the inner and outer parts of the nanopore, thus affecting the electric potential distribution. Therefore, a sufficiently large computational domain is required to reduce the influence of the reservoir resistance on the electric potential distribution of the system.

Considering Case (iv) in Section 3.1 for comparison (a negatively charged nanopore with Joule heating), we simulate nine cases with different reservoir sizes, where the length L_R of the reservoir varies from 200 to 1000 nm. The ion concentration $n_{\pm}$, electric potential ϕ, and temperature $T|_{\text{axis}}$ on the centerline (z-axis: $r = 0, -50 \text{ nm} < z < 50 \text{ nm}$), and temperature $T|_{\text{middle}}$ on the middle line (r-axis: $0 < r < 50 \text{ nm}, z = 0$) of the nanopore are shown in Figure S1a–e. We observe that the changes in the simulation results become smaller as L_R increases, as seen by the results of 800 nm and 1000 nm, which are almost completely overlapped in the figure, such that the simulation result of 1000 nm can be regarded as an accurate convergence result. We define the relative difference in quantity x between the cases of L_R and 1000 nm as $\max\left(\left|\frac{x_{L_R} - x_{1000}}{X}\right| \times 100\%\right)$, where x_{L_R} and x_{1000} are the corresponding values of x on the same coordinate point in the two cases, and X is the reference variation range of x. We consider the ion concentration X as 0.4 M (external solution concentration bias), the electric potential as 2 V (external electric potential difference), and the temperature as 7.15 K (typical temperature change between the midpoint of the nanopore and the system boundary). Under this definition, the relative differences among the simulation results of different L_R and

1000 nm are shown in Figure S1f. The results show that when L_R exceeds 600 nm (not more than 1000 nm), the differences between the simulation results under different L_R conditions are less than 2.5%. Such small differences are the boundary effects that can be considered marginal when the size of the reservoir exceeds 600 nm.

Here, we determine the ion concentration difference $n_+ - n_-$ (proportional to induced charge concentration) and axial velocity v_z on the centerline, and temperature $T|_{\text{axis}}$ and $T|_{\text{middle}}$ in the system for evaluating the quality of different meshes. The results of three meshes with different densities for L_R set as 600 nm are shown in Figure S2, in which mesh 2 doubles the structured cells of mesh 1 (which means mesh 2 covers a larger area surrounding the nanopore using structured cells), and mesh 3 has a higher number of total cells, approximately 1.3 times that of meshes 1 and 2 (detailed information regarding meshes is listed in Table S1). The simulation results indicate that these three meshes are almost identical. In the design of meshes, the influence of the thickness of EDL on the simulation results has been considered already. Usually the characteristic thickness of EDL is measured by Debye length $\lambda_D = \sqrt{\frac{\varepsilon\varepsilon_0 k_{\mathrm{B}} T}{2e^2 n_\infty}}$ for valence-symmetric (1 : 1) electrolytes [18], and $\lambda_D = 0.3$ nm when $T = T_0$, $n_\infty = n_0$. Among all the meshes, there are more than 9 layers of mesh covering the Debye length, and the minimum mesh thickness on the solution side close to the wall of silicon nitride thin layer is 0.02 nm.

Considering all the above factors and for practical concerns, we adopted $L_R = 600$ nm in our simulations, providing accurate results and comparing to the required simulation time when L_R is 800 and 1000 nm; in addition, mesh 2 was adopted, considering that the velocity direction in the nanopore and on the z-axis is almost always parallel to the z-axis.

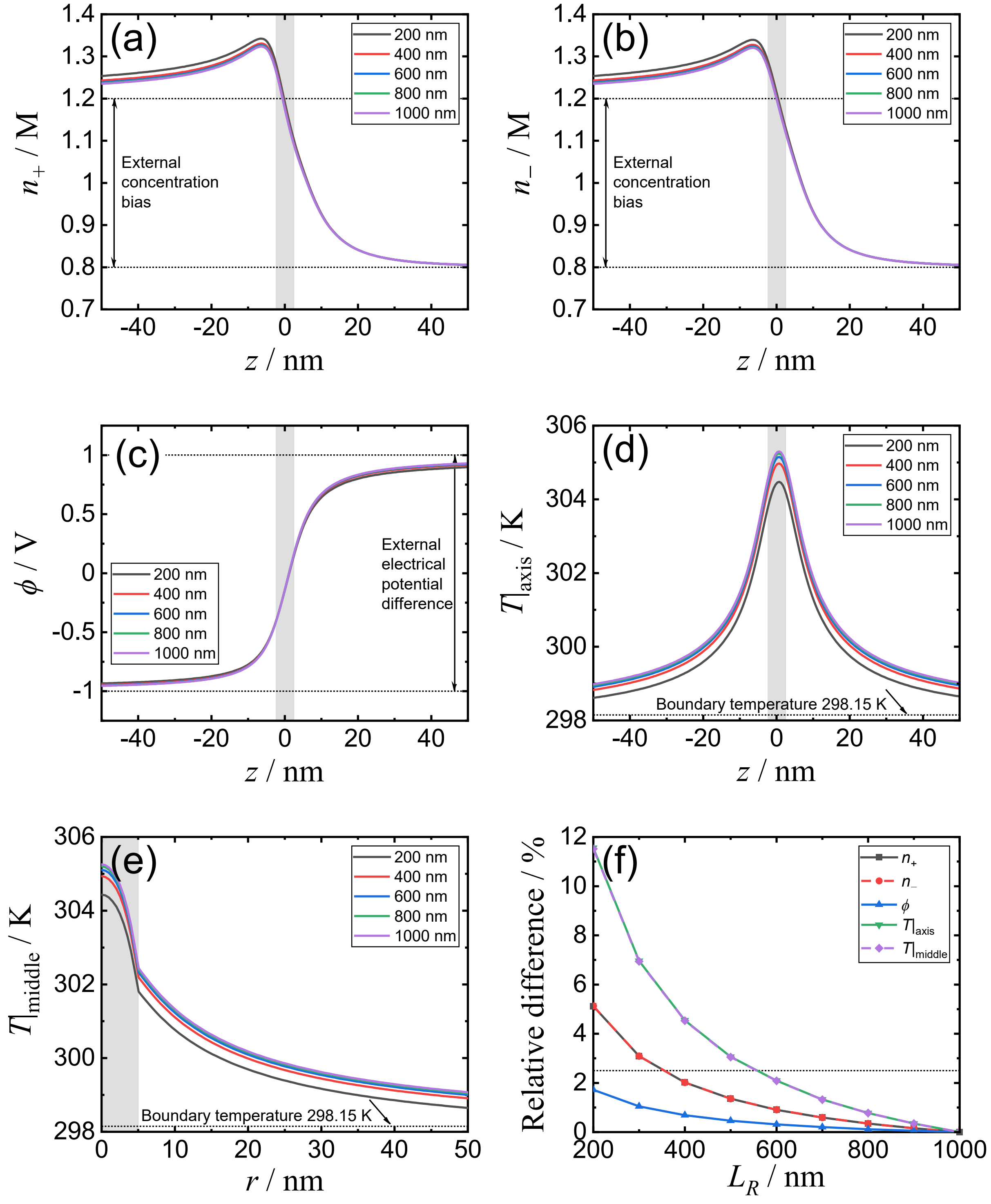

Figure S1 Variations of (**a**) cation and (**b**) anion concentrations, (**c**) electric potential, and (**d**) temperature on the centerline, and (**e**) temperature on the middle line of the nanopore with different reservoir lengths L_R. (**f**) Relative differences among the simulation results of different L_R and 1000 nm. (The shaded part indicates the position of the nanopore.)

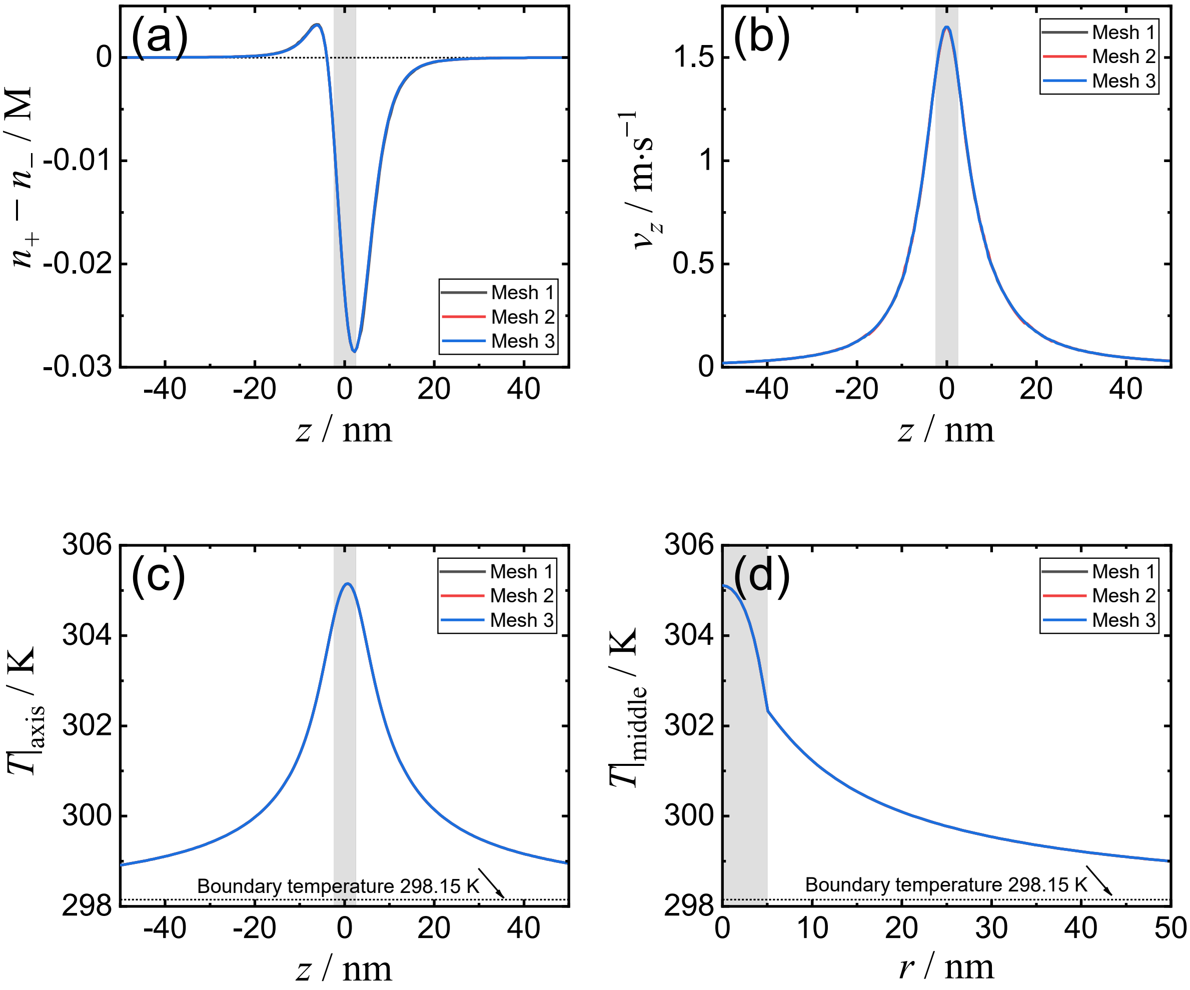

Figure S2 Simulation results of (**a**) ion concentration difference, (**b**) axial velocity, and (**c**) temperature on the centerline, and (**d**) temperature on the middle line of the nanopore with three different meshes when the reservoir length L_R is 600 nm.

Table S1 Mesh number and simulation time.

L_R **of the reservoir** / nm	**600**			**800**	**1000**
	**Mesh 1**	**Mesh 2**	**Mesh 3**		
Number of unstructured (triangle) cells	124 772	100 086	136 432		
Number of structured (quadrangle) cells	21 566	48 918	57 816		
Number of total cells	146 338	149 004	194 248	176 046	202 286
Total CPU time / s	3.5×10^3	3.8×10^3	5.9×10^3	4.8×10^3	6.2×10^3

2. Comparison of the magnitudes of viscous dissipation and Joule heating

In this section, we discuss the heat generated from viscous dissipation $-\eta(\nabla \boldsymbol{v} + (\nabla \boldsymbol{v})^{\mathrm{T}}) : \nabla \boldsymbol{v}$ and that from Joule heating in the nanopore. It was found that the effect of viscous dissipation is far smaller than the Joule heating effects. For instance, for Case (iv) in Section 3.1, when $\Delta\phi = 1, 2,$ and 3 V, the largest ratio of the heat from viscous dissipation and Joule heating inside the nanopore is only $2.0\%, 1.2\%,$ and 0.84% near the nanopore surface, respectively. Therefore, the effect of viscous dissipation is negligible in our model.

3. Information on correlation equations for pure water at 0.1 MPa

The relationships of the water density ρ, viscosity η, static dielectric constant ε, specific isobaric heat capacity c_p, and thermal conductivity κ to temperature T are given by regression equations from [15], which are valid in the temperature range 273.15– 383.15 K for liquid water at 0.1 MPa, as listed in Tables S2–5. These equations are simple but highly accurate compared with the equations from the International Association for the Properties of Water and Steam if only the properties of liquid water at 0.1 MPa are needed.

Table S2 Regression equations for the properties of pure water at the pressure of 0.1 MPa when the temperature is within the range 273.15– 383.15 K. The parameters n, m, a, b, and c are in Table S3; the parameters d, f, g, h, k, and l are in Table S4; other parameters are in Table S5 [15].

Property	Relation
Density	$\rho(T) = \left(\frac{RT_{\mathrm{R}}}{p_0}\left(a_5 + \sum_{i=6}^{10} a_i \alpha^{n_i} + \sum_{i=6}^{10} b_i \beta^{m_i}\right)\right)^{-1}$
Viscosity	$\eta(T)\ /\ (\mathrm{Pa}\cdot\mathrm{s}) = 10^{-6} \cdot \sum_{i=1}^{4} d_i (T^*)^{f_i}$
Static dielectric constant	$\varepsilon(T) = \sum_{i=1}^{4} g_i (T^*)^{h_i}$

Specific isobaric heat capacity	$c_p(T) = -R\left(c_3 + \tau \sum_{i=1}^{3} n_i(n_i+1)a_i\alpha^{n_i+2} + \tau \sum_{i=1}^{4} m_i(m_i+1)b_i\beta^{m_i+2}\right)$
Thermal conductivity	$\kappa(T)\ /\ (\mathrm{W\cdot m^{-1}\cdot K^{-1}}) = \sum_{i=1}^{4} k_i(T^*)^{l_i}$

Table S3 Parameters n, m, a, b, and c for the regression equations in Table S2.

i	n_i	m_i	a_i	b_i	c_i
1	4	2	$-1.661\,470\,539 \times 10^{5}$	$-8.237\,426\,256 \times 10^{-1}$	
2	5	3	$2.708\,781\,640 \times 10^{6}$	$1.908\,956\,353$	
3	7	4	$-1.557\,191\,544 \times 10^{8}$	$-2.017\,597\,384$	$-8.983\,025\,854$
4		5		$8.546\,361\,348 \times 10^{-1}$	
5		1	$1.937\,631\,57 \times 10^{-2}$	$5.785\,452\,92 \times 10^{-3}$	
6	4	2	$6.744\,584\,46 \times 10^{3}$	$-1.531\,956\,65 \times 10^{-2}$	
7	5	3	$-2.225\,216\,04 \times 10^{5}$	$3.113\,378\,59 \times 10^{-2}$	
8	7	4	$1.002\,312\,47 \times 10^{8}$	$-4.235\,462\,41 \times 10^{-2}$	
9	8	5	$-1.635\,521\,18 \times 10^{9}$	$3.387\,135\,07 \times 10^{-2}$	
10	9	6	$8.322\,996\,58 \times 10^{9}$	$-1.199\,467\,61 \times 10^{-2}$	

Table S4 Parameters d, f, g, h, k, and l for the regression equations in Table S2.

i	1	2	3	4
d_i	280.68	511.45	61.131	0.459 03
f_i	−1.9	−7.7	−19.6	−40.0
g_i	−43.7527	299.504	−399.364	221.327
h_i	−0.05	−1.47	−2.11	−2.31
k_i	0.802 01	−0.259 92	0.100 24	−0.032 005
l_i	−0.32	−5.7	−12.0	−15.0

Table S5 Other parameters for the regression equations in Table S2.

	Expression	Value
R		$461.518\ 05\ \mathrm{J \cdot kg^{-1} \cdot K^{-1}}$
T_R		$10\ \mathrm{K}$
p_0		$0.1\ \mathrm{MPa}$
T_a		$593\ \mathrm{K}$
T_b		$232\ \mathrm{K}$
α	$\frac{T_\mathrm{R}}{T_\mathrm{a} - T}$	
β	$\frac{T_\mathrm{R}}{T - T_\mathrm{a}}$	
T^*	$\frac{T}{300\ \mathrm{K}}$	
τ	$\frac{T}{T_\mathrm{R}}$	
